# Real-World Evaluation of the Sysmex XN-9100 WDF–WPC Workflow: Diagnostic Performance, False-Negative Burden, and Potential Optimization of Smear Review Workload

**DOI:** 10.3390/diagnostics16152387

**Published:** 2026-07-29

**Authors:** Francesca Romano, Domenico Romeo, Sara Ciullini Mannurita, Edda Russo, Eva Milletti, Alessandra Fanelli, Alessandro Bonari

**Affiliations:** 1 General Laboratory, Azienda Ospedaliero-Universitaria Careggi, 50134 Florence, Italy; romanofr@aou-careggi.toscana.it (F.R.); domenico.romeo@unifi.it (D.R.); millettie@aou-careggi.toscana.it (E.M.); fanellia@aou-careggi.toscana.it (A.F.); bonaria@aou-careggi.toscana.it (A.B.); 2 Clinical Pathology Laboratory Unit, S. Giuseppe Hospital, Azienda USL Toscana Centro, 50053 Empoli, Italy

**Keywords:** Sysmex XN-9100, WDF channel, WPC channel, false-negative burden, reflex testing, diagnostic performance, workflow efficiency, peripheral blood smear

## Abstract

**Background:** Modern hematology analyzers integrate multiparametric technologies and adaptive flagging algorithms to support rapid detection of abnormal leukocyte populations. The Sysmex XN-9100 employs the White Blood Cell Differential (WDF) channel for first-line screening and the White Precursor Cell (WPC) channel as a reflex test for improved detection of blasts and abnormal lymphocytes. Although widely implemented, the real-world diagnostic performance and workflow implications of this integrated strategy remain incompletely characterized. **Objectives:** To evaluate, in a large real-world cohort, the performance of the Sysmex XN-9100 WDF–WPC workflow, focusing on its diagnostic accuracy, its capability to identify pathological cases within the WDF-negative population, and its potential implications for blood smear review workload optimization. **Methods:** A retrospective analysis was performed on 4531 consecutive routine blood samples reviewed between February and June 2025. All samples were analyzed using the Sysmex XN-9100. WDF-positive cases (“Blasts/Abnormal lymphocytes?”) underwent reflex WPC testing (“Blasts?” and “Abnormal lymphocytes?”). Peripheral blood smear morphology, based on the evaluation of 200 leukocytes using the DI-60 digital imaging system, constituted the reference standard. Diagnostic performance metrics, including sensitivity, specificity, predictive values, likelihood ratios, accuracy, and ROC analysis, were calculated for WDF and WPC channels using both individual and aggregated flag evaluations. **Results:** The WDF-B/AL (White Blood Cell Differential Channel-“Blasts/Abnormal lymphocytes?”) flag demonstrated high sensitivity (88.24%) and negative predictive value (92.73%), supporting its role as an effective first-line screening tool, although specificity was limited (48.87%; AUC = 0.75). Unlike most previous studies, our workflow allowed evaluation of pathological cases occurring within the WDF-negative population, providing a realistic estimate of the false-negative burden. Disaggregated WPC analysis revealed complementary diagnostic profiles: WPC-B (White Precursor Cell Channel-“Blasts?”) showed higher specificity (77.71%) and overall discriminative performance (AUC = 0.81), whereas WPC-AL (White Precursor Cell Channel-“Abnormal lymphocytes?”) achieved higher sensitivity (92.18%) and negative predictive value (95.99%). Aggregated evaluation of WPC flags (logical OR) maximized sensitivity (92.68%) and reduced false-negative cases, supporting the role of the WPC channel as a second-level diagnostic safety net. Furthermore, within the integrated laboratory workflow, WPC-negative results may support the optimization of manual smear-review allocation when interpreted together with predefined institutional smear-review criteria, while maintaining diagnostic safety. **Conclusions:** The Sysmex XN-9100 WDF–WPC workflow constitutes a multilayered high-sensitivity screening strategy that prioritizes abnormality detection while optimizing laboratory efficiency. The complementary behavior of WPC-B and WPC-AL enhances overall workflow safety, whereas evaluation of WDF-negative pathological cases provides a more realistic assessment of screening performance than conventional reflex-based studies. Beyond analytical accuracy, the workflow may contribute to balancing diagnostic reliability, patient safety, and resource utilization in high-volume laboratory practice.

## 1. Introduction

Complete blood count (CBC) with leukocyte differential is one of the most frequently requested laboratory tests in clinical practice and plays a pivotal role in the initial assessment of hematological and systemic disorders [1,2]. Over recent decades, the transition from manual cell counting to fully automated hematology analyzers has substantially improved analytical precision, standardization, and turnaround times, while enabling the implementation of advanced flagging systems designed to identify potentially abnormal leukocyte populations at an early stage [1,3,4].

Modern automated hematology analyzers employ different technological approaches—including fluorescence flow cytometry, multi-angle light scatter, impedance, and cytochemical methods—to support automated leukocyte differentiation and abnormal cell detection. Comparative studies have demonstrated that, despite differences in analytical principles among platforms, overall analytical performance is broadly comparable, whereas the efficiency of abnormal cell flagging may vary across manufacturers and algorithms [5,6,7,8,9]. Within the Sysmex XN-series, the WDF channel acts as the primary screening level, whereas the WPC channel is reflexively activated to further investigate samples suspected of containing blasts or abnormal lymphocytes [10,11]. Together, these channels constitute a sequential screening strategy aimed at maximizing the detection of clinically relevant hematological abnormalities while limiting unnecessary microscopic blood smear reviews [7,12].

Despite their widespread implementation, several aspects of the real-world performance of WDF and WPC remain incompletely understood. Most published studies have evaluated these channels within predefined manufacturer-based workflows, in which WDF-positive samples undergo reflex WPC testing, whereas WDF-negative samples are generally not subjected to systematic reassessment [8,13,14,15]. Consequently, the actual burden of false-negative WDF results remains largely unknown, and the true screening performance of the first-level flagging system may be difficult to estimate. This limitation is particularly relevant in routine laboratory practice, where additional review criteria, alternative analyzer flags, or clinical considerations may still lead to microscopic examination of samples initially classified as negative.

Another unresolved issue concerns the role of the WPC channel within the overall diagnostic workflow. Although it is generally regarded as a second-level tool designed to improve specificity, published studies have reported highly heterogeneous results, with substantial variability in sensitivity and specificity estimates across different populations and laboratory settings [8,13,14,16]. Furthermore, most available investigations focus on the overall performance of the WPC channel without separately evaluating the contribution of the “Blasts?” and “Abnormal lymphocytes?” flags, making it difficult to understand their individual diagnostic behavior and their respective contribution to workflow performance [15,17]. Whether the principal value of the WPC channel lies in improving specificity or in providing an additional safety layer within the screening process remains unclear.

Peripheral blood smear examination remains the reference method for confirming the presence of abnormal leukocyte populations, particularly blasts and atypical lymphocytes [18,19]. However, manual review is labor-intensive, time-consuming, and subject to inter-observer variability. Therefore, optimizing automated screening strategies is not only a diagnostic issue, but also an important organizational challenge for modern high-volume laboratories [4,18].

In this context, the present study was designed to provide a comprehensive real-world evaluation of the Sysmex XN-9100 WDF–WPC workflow. Unlike most previous studies, our laboratory setting allowed pathological cases occurring within the WDF-negative population to be identified through independent smear-review criteria, providing a unique opportunity to estimate the true false-negative burden of the WDF screening process and to assess the real-world reliability of first-level automated screening. In addition, we evaluated the WPC channel using both disaggregated (WPC-B and WPC-AL) and aggregated approaches to characterize not only the performance of individual flags but also the complementary contribution of the two signals to the overall diagnostic workflow. Finally, we assessed the potential implications of the reflex strategy for smear-review workload allocation, thereby integrating diagnostic performance with considerations of real-world laboratory efficiency.

## 2. Materials and Methods

### 2.1. Sample Collection

The study included samples analyzed as part of the routine laboratory workflow between 1 February and 30 June 2025. During this period, 75,882 complete blood counts (CBCs) with differential leukocyte counts were performed.

A total of 4531 samples were included and reviewed by a single hematologist with extensive expertise in peripheral blood smear examination. The study population comprised adult patients aged ≥20 years, reflecting the referral pathway of our tertiary university hospital, which exclusively manages adults; patients younger than 20 years are referred to the regional pediatric referral center. No upper age exclusion criterion was applied.

Blood samples were collected in K3-EDTA Vacutainer™ tubes (Becton Dickinson, Franklin Lakes, NJ, USA) following standard pre-analytical protocols and processed within 6 h of collection using Sysmex XN-9100 hematology analyzers (Sysmex Corporation, Kobe, Japan). Instrument Q-flag and alarm settings were kept at the manufacturer’s default values. Furthermore, an institution-specific rule engine was implemented to prevent autoverification of selected samples based on predefined complete blood count parameter thresholds, patient longitudinal laboratory history, and test priority (routine vs. urgent), thereby directing these specimens to manual expert review.

Data were retrospectively collected and organized into three CSV files: (1) CBC and differential parameters; (2) morphological comments from blood smear review; (3) instrument-derived Q-flag data.

### 2.2. Description of the Operational Workflow

**Figure 1:**
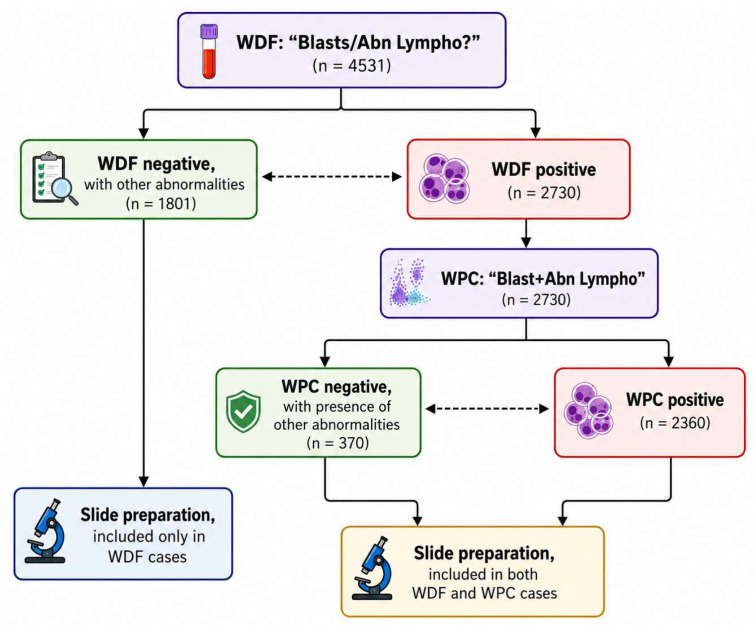
Study workflow and sample selection process. A total of 4531 samples were evaluated using the WDF channel (“Blasts/Abnormal lymphocytes?”). Of these, 1801 samples were WDF-negative but met additional smear-review criteria due to the presence of other abnormalities and were therefore included exclusively in the WDF dataset. The remaining 2730 WDF-positive samples underwent reflex analysis using the WPC channel. Among these, 2360 samples were WPC-positive, whereas 370 were WPC-negative but fulfilled additional review criteria because of other abnormalities. Samples requiring microscopic examination were subjected to peripheral blood smear preparation and review. WDF-positive samples contributed to both the WDF and WPC datasets, while WDF-negative samples with alternative review triggers contributed only to the WDF dataset. Green boxes indicate negative screening results, red boxes indicate positive screening results, and purple boxes represent analyzer screening flags. Dashed arrows denote complementary screening outcomes, while microscope icons indicate cases selected for peripheral blood smear preparation.

All samples underwent initial leukocyte differential analysis on the WDF channel. The presence of the “Blasts/Abnormal lymphocytes?” flag, which was the focus of the present study, was evaluated. Samples positive for this flag were automatically reflex-tested on the WPC channel, where the “Blasts?” and “Abnormal lymphocytes?” flags were assessed. If at least one WPC flag was positive, a digital peripheral blood smear was automatically generated using the DI-60 digital cell imaging analyzer (CellaVision AB, Lund, Sweden). For each sample, 200 leukocytes were analyzed, and the acquired images were reviewed by a single experienced hematologist. Morphological findings, particularly the presence of atypical lymphocytes and the percentage of blasts, were recorded and used as the reference standard for evaluating the diagnostic performance of the instrument-generated flags (Figure 1).

Both WDF and WPC channels generate other flags related to different abnormalities, including severe neutropenia (absolute neutrophil count < 0.5 × 10^3^/µL), severe anemia (hemoglobin < 6.5 g/dL), thrombocytopenia (platelet count < 100 × 10^3^/µL), as well as abnormalities reflecting atypical distribution or separation of cellular populations within the scattergram analysis. Although these flags could trigger peripheral blood smear review and the corresponding samples were included in the appropriate dataset, they were not evaluated further because they were outside the primary focus of the present study.

Samples without specific positivity for the flags under investigation, despite the presence of other abnormalities, were classified as negative for the study-specific flags. This approach allowed us to examine two distinct datasets:

- One dataset analyzing the performance of the WDF channel alone.
- One dataset analyzing the performance of the WPC channel in cases with a positive WDF flag.

Samples requiring smear review because of these additional flags were also included in the corresponding channel analysis. When the study-specific flags (“Blasts/Abnormal lymphocytes?”, “Blasts?”, or “Abnormal lymphocytes?”) were not present, these samples were classified as negative for the purposes of the present analysis. The use of a single experienced hematologist ensured consistency of morphological interpretation and minimized inter-observer variability.

### 2.3. Evaluation of Instrument Flags and Microscopic Blood Smears

The flags “Blasts/Abnormal lymphocytes?”, “Blasts?”, and “Abnormal lymphocytes?” were evaluated using a numerical scale (Q-value) generated by the Sysmex XN-9100 analyzer. The Q-value ranges from 0 to 300, increasing in increments of 10 units.

For the determination of positivity, the manufacturer’s standard settings were applied: a flag was considered positive when the Q-value was ≥100.

For each sample, 200 leukocytes were analyzed on peripheral blood smears using the DI-60 digital cell imaging analyzer. The acquired images were subsequently reviewed and reported by a single experienced hematologist according to the criteria established by the International Society for Laboratory Hematology (ISLH) guidelines. The hematologist performing the morphological evaluation was not blinded to the analyzer-generated flags, as the review was conducted according to the routine laboratory workflow, in which instrument information is available to support morphological assessment and clinical decision-making. Morphological positivity was defined, for the purposes of the present study, as the identification of any morphologically recognizable blast population or atypical lymphocyte, with no minimum percentage threshold required. The detection of any blast or atypical lymphocyte within the reviewed smear was considered sufficient to classify the sample as positive for the corresponding category. In cases of uncertain or borderline morphological interpretation, findings were reported as comments in the hematological report rather than classified as definitively positive or negative. When clinically appropriate, additional second-level investigations, such as immunophenotypic analysis by flow cytometry, were recommended. Morphological findings, including the presence of blasts and atypical lymphocytes, were recorded and used as the morphological reference standard for assessing the diagnostic performance of the instrument-generated flags.

### 2.4. Data Extraction, Processing, and Statistical Analysis

Three CSV files were initially generated, displaying structural differences. A Python [20] algorithm implemented in Visual Studio Code [21] was used to harmonize the datasets and create a single CSV file containing all necessary information.

For both WDF and WPC samples, diagnostic performance was assessed in Microsoft Excel [22] by calculating the following parameters: sensitivity, specificity, positive predictive value (PPV), negative predictive value (NPV), accuracy, and positive and negative likelihood ratios (LR+ and LR−).

ROC (Receiver Operating Characteristic) curve analysis was performed using Python to evaluate discriminative performance through the area under the curve (AUC), and the Youden index was calculated to identify the optimal cut-off maximizing both sensitivity and specificity.

WPC data were analyzed using two approaches:

- Disaggregated data: the two WPC flags, “Blasts?” and “Abnormal lymphocytes?”, were evaluated separately, each considered positive independently.
- Aggregated data: overall WPC positivity was defined using a logical OR; the channel was considered positive if at least one flag was positive, and negative only if both flags were negative.

## 3. Results

### 3.1. Evaluation of the WDF Channel Flag

**Figure 2:**
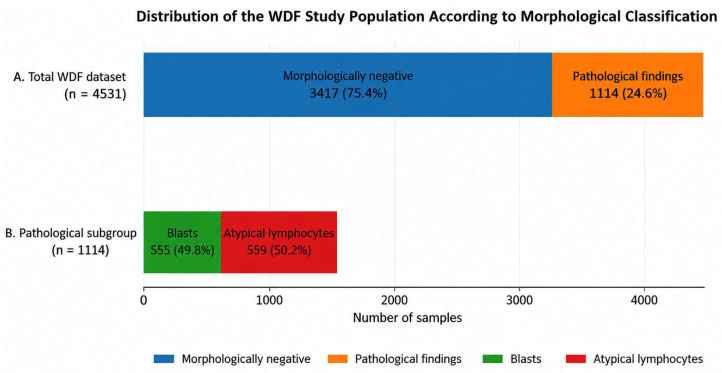
Distribution of the WDF study population according to morphological classification. (**A**) Overall distribution of the 4531 samples included in the WDF dataset. A total of 3417 samples (75.41%) were morphologically negative, whereas 1114 samples (24.59%) showed pathological findings. (**B**) Composition of the pathological subgroup. Among the 1114 pathological samples, 555 (49.82%) contained blasts and 559 (50.18%) contained atypical lymphocytes.

The WDF dataset was evaluated according to the microscopic review of peripheral blood smears (Figure 2). Among the 4531 samples analyzed, 1114 (24.59%) showed pathological findings, including 555 samples (12.25%) containing blasts and 559 samples (12.34%) containing atypical lymphocytes. The remaining 3417 samples (75.41%) were morphologically negative.

Based on the WDF-B/AL flag, 2730 samples (60.25%) were classified as positive and 1801 (39.75%) as negative (Table 1).

Among the 1801 WDF-B/AL− samples, 1670 were confirmed negative by microscopic review, whereas 131 showed pathological findings, including 109 blast-positive and 22 atypical lymphocyte-positive samples. Among the 2730 WDF-B/AL+ samples, 446 contained blasts, 537 contained atypical lymphocytes, and 1747 were morphologically negative.

The confusion matrix analysis yielded 983 true positives (TP), 1747 false positives (FP), 1670 true negatives (TN), and 131 false negatives (FN) (Table 2).

Diagnostic performance metrics are summarized in Table 3. The WDF-B/AL flag showed a sensitivity of 88.24% (95% CI: 86.20–90.07), specificity of 48.87% (95% CI: 47.18–50.56), positive predictive value (PPV) of 36.01% (95% CI: 34.20–37.84), and negative predictive value (NPV) of 92.73% (95% CI: 91.43–93.88). Overall accuracy was 58.55% (95% CI: 57.10–59.99), with LR+ and LR− values of 1.73 and 0.24, respectively.

**Figure 3:**
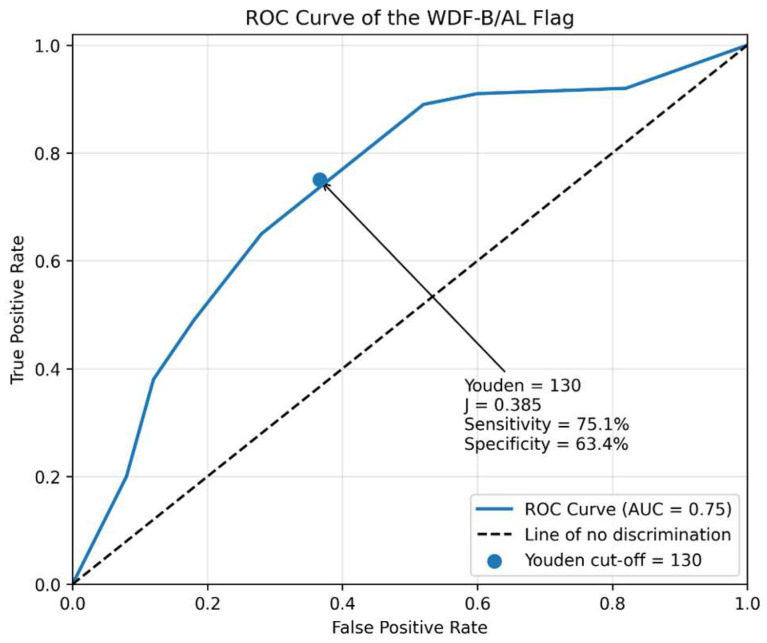
Receiver Operating Characteristic (ROC) curve of the WDF-B/AL flag for the detection of blasts and abnormal lymphocytes. The ROC analysis yielded an area under the curve (AUC) of 0.75, indicating good overall discriminative performance of the WDF-B/AL screening flag. The arrow indicates the optimal cutoff value, identified using the Youden index, which was 130 (Youden index = 0.385), corresponding to a sensitivity of 75.1% and a specificity of 63.4%, as indicated by the highlighted point on the curve. The dashed diagonal line represents the performance expected by random classification (AUC = 0.50).

ROC analysis yielded an AUC of 0.75. The Youden-derived optimal cut-off was 130, corresponding to a sensitivity of 75.1% and a specificity of 63.4% (Figure 3).

### 3.2. Evaluation of the WPC Channel Flags

According to the study workflow (Figure 1), all samples positive for the WDF-B/AL flag underwent reflex analysis using the WPC channel.

**Figure 4:**
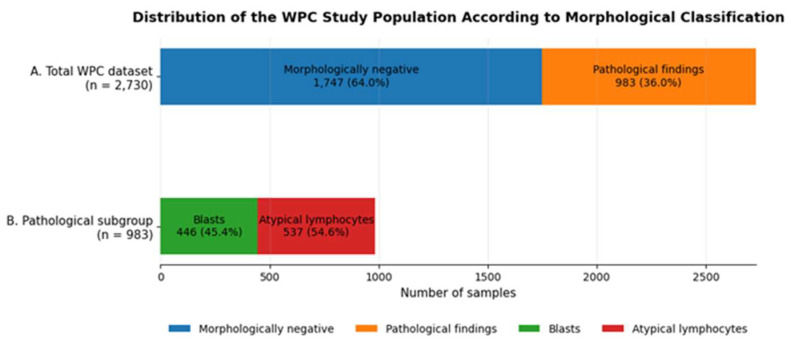
Distribution of the WPC study population according to morphological classification. (**A**) Overall distribution of the 2730 samples included in the WPC dataset. A total of 1747 samples (63.99%) were morphologically negative, whereas 983 samples (36.01%) showed pathological findings. (**B**) Composition of the pathological subgroup. Among the 983 pathological samples, 446 (45.37%) contained blasts and 537 (54.63%) contained atypical lymphocytes, as determined by peripheral blood smear review.

Among the 2730 samples analyzed by WPC, 983 (36.01%) showed pathological findings, including 446 blast-positive samples (16.34%) and 537 atypical lymphocyte-positive samples (19.67%). The remaining 1747 samples (63.99%) were morphologically negative (Figure 4).

Overall, the WPC channel generated 678 “Blasts?” flags (24.84%), 1525 “Abnormal lymphocytes?” flags (55.86%), 157 combined “Blasts? + Abnormal lymphocytes?” flags (5.75%), and 370 negative results (13.55%) (Table 4).

#### 3.2.1. Disaggregated Analysis

Confusion matrices for WPC-B and WPC-AL are reported in Table 5 and Table 6, respectively, whereas the corresponding diagnostic performance metrics are summarized in Table 7.

For blast detection, WPC-B generated 326 TP, 509 FP, 1775 TN, and 120 FN. Diagnostic performance included a sensitivity of 73.09%, specificity of 77.71%, PPV of 39.04%, and NPV of 93.67%.

For atypical lymphocyte detection, WPC-AL generated 495 TP, 1187 FP, 1006 TN, and 42 FN. Diagnostic performance included a sensitivity of 92.18%, specificity of 45.87%, PPV of 29.43%, and NPV of 95.99%.

**Figure 5:**
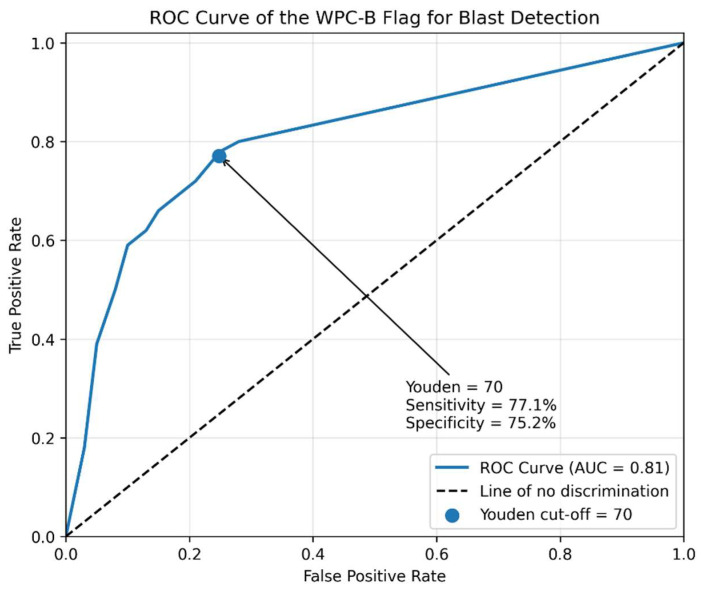
Receiver Operating Characteristic (ROC) curve of the WPC-B flag for the detection of blasts. The ROC analysis yielded an area under the curve (AUC) of 0.81, indicating good discriminative performance of the WPC-B flag. The optimal threshold identified by the Youden index was 70, corresponding to a sensitivity of 77.1% and a specificity of 75.2%, as indicated by the highlighted point on the curve marked by the arrow. The dashed diagonal line represents the line of no discrimination (AUC = 0.50).

**Figure 6:**
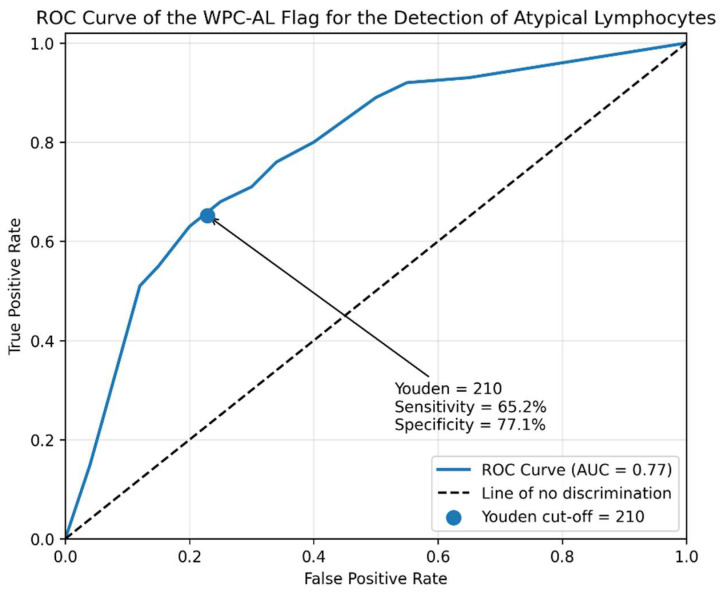
Receiver Operating Characteristic (ROC) curve of the WPC-AL flag for the detection of abnormal lymphocytes. The ROC analysis yielded an area under the curve (AUC) of 0.77, indicating acceptable discriminative performance of the WPC-AL flag. The optimal threshold identified by the Youden index was 210, corresponding to a sensitivity of 65.2% and a specificity of 77.1%, as indicated by the highlighted point on the curve marked by the arrow. The dashed diagonal line represents the line of no discrimination (AUC = 0.50).

ROC analysis demonstrated an AUC of 0.81 for WPC-B, with a Youden-derived cut-off of 70 corresponding to a sensitivity of 77.1% and a specificity of 75.2% (Figure 5). For WPC-AL, the AUC was 0.77, with a Youden-derived cut-off of 210 corresponding to a sensitivity of 65.2% and a specificity of 77.1% (Figure 6).

#### 3.2.2. Aggregated Analysis

For the aggregated WPC analysis, WPC-B and WPC-AL were combined using a logical OR approach. A sample was considered positive if at least one of the two flags was activated.

The confusion matrix yielded 911 TP, 1449 FP, 298 TN, and 72 FN (Table 8).

Diagnostic performance metrics are reported in Table 9. The aggregated WPC approach achieved a sensitivity of 92.68% (95% CI: 90.80–94.23), specificity of 17.06% (95% CI: 15.32–18.91), PPV of 38.60% (95% CI: 36.63–40.60), and NPV of 80.54% (95% CI: 76.13–84.45). Overall accuracy was 44.29% (95% CI: 42.41–46.17), with LR+ and LR− values of 1.12 and 0.43, respectively.

## 4. Discussion

In routine laboratory practice, the WDF channel represents the first analytical level for detecting potential lymphoid abnormalities. As a screening tool, its primary objective is to identify patients who may harbor clinically relevant hematological abnormalities while minimizing false-negative results. Since no manufacturer-declared reference values for WDF sensitivity and specificity are available, its performance is primarily interpreted in light of published studies, most of which evaluate WDF together with reflex WPC testing [8,13,14,16]. In these studies, WDF-negative samples are generally not reassessed, preventing direct estimation of the false-negative burden of first-level screening.

A major strength of our study lies in the organizational structure of our laboratory workflow. Beyond the WDF-B/AL flag, additional review criteria may trigger microscopic smear examination, including other abnormal lymphocyte-related flags and predefined smear-review rules. This setting allowed pathological cases occurring within the WDF-negative population to be retrieved and evaluated. Importantly, this approach enabled not only the evaluation of flagged pathological samples but also the identification of abnormalities occurring within the WDF-negative population, providing a unique opportunity to estimate the true false-negative burden of the first-level screening process. The absence of pediatric patients represents a limitation of the present study, as the performance and clinical relevance of hematological analyzer flags may differ in younger populations.

Our findings indicate that WDF-B/AL functions effectively as a first-line screening tool. The high sensitivity (88.24%), elevated negative predictive value (92.73%), and low negative likelihood ratio (LR− = 0.24) support its ability to reliably exclude disease when the flag is absent. These results are consistent with the intended screening role of the WDF channel and broadly align with observations reported in previous studies [8,13,14,16]. In our dataset, 131 false-negative cases were identified among 4531 reviewed samples, representing 2.89% of all reviewed samples. According to the conventional definition, the false-negative rate was 11.76% (131/1114). Conversely, the moderate-to-low specificity (48.87%), limited positive predictive value (36.01%), and modest positive likelihood ratio (LR+ = 1.73) indicate that WDF-B/AL should not be considered a confirmatory test. The high number of false-positive results (1747 cases; 38.56%) further reinforces its role as a highly sensitive first-level screening filter rather than a diagnostic confirmation tool.

ROC curve analysis yielded an AUC of 0.75, indicating good overall discriminative ability. The Youden index identified an optimal cut-off value of 130, corresponding to a sensitivity of 75.1% and a specificity of 63.4%. The Youden index identifies the threshold that maximizes the combined contribution of sensitivity and specificity. Interestingly, the currently adopted threshold is lower than the Youden-derived cut-off and therefore prioritizes sensitivity over specificity. In our dataset, this choice translated into a sensitivity increase of 13.14 percentage points compared with the Youden threshold. From a clinical perspective, this finding supports the appropriateness of the current setting, where maximizing case detection is preferable to reducing false-positive alerts.

The possibility of evaluating false-negative samples provided additional insights into the analytical behavior of the WDF-B/AL flag. A closer examination of missed cases revealed that blasts were more frequently overlooked than atypical lymphocytes (109 versus 22 cases, respectively), suggesting differential performance according to the underlying pathological population. Interestingly, this pattern was consistently observed across both WDF and WPC analyses, suggesting that blast-containing samples may represent a more challenging analytical target than atypical lymphocytes within the automated classification system. This observation highlights the importance of maintaining complementary review criteria within the laboratory workflow, allowing pathological samples that may escape first-level automated screening to be identified through alternative pathways.

The WPC channel represents the second analytical level of the Sysmex workflow and is reflexively activated for samples testing positive on the WDF channel. According to the manufacturer, the WPC channel is intended to improve the overall specificity of the diagnostic pathway compared with WDF alone [12]. In principle, this second-level analysis may help refine the selection of suspicious samples and support the allocation of smear-review resources while maintaining overall detection capability through a sequential evaluation process.

However, the literature reports heterogeneous results regarding WPC performance. Studies evaluating the WPC channel in aggregate, considering positivity of either WPC-B or WPC-AL, have reported sensitivity values ranging from 97% to 99% and specificity values between 29% and 88.3% [14,17]. Flag-specific analyses have shown sensitivities of 88.2–100% and specificities of 33.7–85.5% for blast detection [8,13,16], while sensitivities of 72–100% and specificities of 59.6–96.2% have been reported for atypical lymphocyte detection [8,13,16]. Such variability likely reflects differences in study design, patient populations, review criteria, and local laboratory workflows.

Interestingly, our findings suggest that, within our laboratory setting, the principal contribution of the WPC channel is not an increase in specificity, but rather an enhancement of the overall sensitivity and safety of the diagnostic workflow. This effect becomes particularly evident when the channel is evaluated both at the individual-flag level and as an integrated screening system.

WPC-B demonstrated a sensitivity of 73.09%, a negative predictive value (NPV) of 93.67%, and a negative likelihood ratio (LR−) of approximately 0.35, indicating a satisfactory ability to exclude blast-containing samples when the flag is absent. Overall, the channel correctly identified 326 of 446 morphologically confirmed blast-positive cases, whereas 120 cases (26.9%) were not flagged by WPC-B. However, these false-negative results should not necessarily be interpreted as failures of the overall WPC workflow. Indeed, a proportion of blast-containing samples were assigned to alternative WPC categories, particularly WPC-AL, indicating that blast detection is not exclusively dependent on the WPC-B flag. This observation highlights the complementary nature of the WPC algorithm and suggests that the clinical utility of the channel is better represented by the aggregated WPC response than by the performance of individual flags considered in isolation.

The specificity of WPC-B was 77.71%, the highest among the evaluated flags, confirming its greater ability to restrict positivity to samples with a higher probability of containing blasts. Nevertheless, the relatively low positive predictive value (PPV = 39.04%) and modest positive likelihood ratio (LR+ = 3.28) indicate limited confirmatory power. The ROC analysis yielded an AUC of 0.81, reflecting the best overall discriminative performance among the evaluated flags. The Youden-derived cut-off was identified at 70, corresponding to a sensitivity of 77.1% and a specificity of 75.2%, suggesting that the currently adopted threshold already operates close to the optimal balance between sensitivity and specificity.

Interestingly, comparison with WPC-AL revealed distinct but complementary diagnostic profiles. Whereas WPC-B favored specificity, accuracy, and overall discrimination, WPC-AL prioritized sensitivity, negative predictive value, and rule-out capability. This complementary behavior suggests that the two flags should not be interpreted as independent diagnostic tools but rather as synergistic components of the same screening strategy. Indeed, some blast-containing samples not identified by WPC-B were captured by alternative WPC categories, particularly WPC-AL, indicating that the clinical utility of the WPC channel extends beyond the performance of individual flags. This observation also explains why evaluation of the WPC channel at the workflow level may be more informative than the isolated assessment of individual flags and provides a rationale for the superior performance observed in the aggregated WPC analysis.

In contrast, WPC-AL displayed a profile more closely resembling that of a screening test. Sensitivity reached 92.18%, NPV was 95.99%, and LR− was 0.17, indicating strong rule-out capability. However, specificity remained limited (45.87%), with low PPV (29.43%) and weak LR+ (1.7). The AUC of 0.77 indicated acceptable discriminative performance, although slightly lower than that observed for WPC-B. Notably, the Youden-derived threshold of 210 differed substantially from the currently implemented cut-off, suggesting that the present configuration strongly favors sensitivity over specificity.

While disaggregated analysis provides important information regarding the behavior of individual flags, the aggregated evaluation may better reflect real-world laboratory practice. From an operational perspective, the clinically relevant question is not whether blasts or atypical lymphocytes are identified separately, but whether any potentially significant abnormality is detected and appropriately routed for further investigation. The rationale for this aggregated approach is supported by the complementary behavior of the two WPC flags. Whereas WPC-B favors specificity and overall discrimination, WPC-AL prioritizes sensitivity and exclusion of pathological cases. Consequently, samples missed by one flag may still be captured by the other, creating a multilayered detection system that enhances the overall safety of the workflow.

Accordingly, when WPC-B and WPC-AL were evaluated as a combined flag using a logical OR approach, the channel achieved a sensitivity of 92.68%, with only 72 false-negative cases (2.64%). Although specificity decreased substantially (17.06%), this finding should be interpreted in light of the intended purpose of the aggregated WPC strategy. Rather than improving diagnostic confirmation, the combined approach is designed to maximize abnormality detection. Indeed, the number of false-negative cases was markedly reduced compared with the individual flag analyses, demonstrating the complementary behavior of WPC-B and WPC-AL.

Beyond its analytical performance, the WPC channel may provide operational advantages within the integrated laboratory workflow. In our dataset, the 370 WPC-negative samples represented cases in which the WPC channel did not generate a positive flag. However, these samples were not necessarily exempted from manual smear review, as additional institution-specific rules could independently trigger microscopic examination. These criteria included severe neutropenia (absolute neutrophil count < 0.5 × 10^3^/µL), severe anemia (hemoglobin < 6.5 g/dL), thrombocytopenia (platelet count < 100 × 10^3^/µL), as well as abnormalities related to atypical distribution or separation of cellular populations in scattergram analysis.

Accordingly, the 72 morphologically positive cases identified among the WPC-negative samples were reviewed because of these additional laboratory criteria and were not missed by the overall diagnostic workflow. Therefore, the WPC channel should be interpreted as an additional layer within a multimodal screening strategy rather than as a standalone criterion for smear exclusion. When considered within this integrated approach, WPC results may support the optimization of smear-review allocation when interpreted together with independent institutional review criteria. This finding highlights the potential role of the WDF–WPC workflow in supporting workload and resource allocation optimization in routine laboratory practice, where efficient use of resources is increasingly important.

Compared with our previous study [23] performed in a low-prevalence cohort of healthy blood donors, the present work addresses a different clinical question by evaluating the WDF–WPC reflex workflow in consecutive routine clinical samples, allowing assessment of false-negative cases and workflow optimization in real-world laboratory practice.

Overall, our findings suggest that the value of the integrated WDF–WPC workflow lies not primarily in increasing specificity, but in providing a multilayered screening strategy capable of maximizing abnormality detection while maintaining diagnostic safety through the integration of automated flagging and institution-specific smear-review criteria. The present study reflects the routine workflow of our laboratory, in which all CBCs performed during the study period were processed according to standard institutional practice without any pre-selection for research purposes. The analytical evaluation focused on consecutive smear-reviewed samples and, specifically, on cases with WDF-B/AL positivity undergoing reflex WPC analysis. Therefore, the reported diagnostic performance should be interpreted within the context of this real-world diagnostic pathway and not as representative of the entire routine CBC population.

## 5. Conclusions

This study provides a real-world evaluation of the Sysmex XN-9100 WDF–WPC workflow within routine laboratory practice. The integrated WDF–WPC pathway showed high sensitivity for the detection of blasts and abnormal lymphocytes, supporting its role as a multilayered screening strategy rather than as a confirmatory diagnostic approach.

Within the evaluated workflow, the main contribution of the WPC channel was the enhancement of overall screening safety rather than an increase in diagnostic specificity. The complementary behaviour of the WPC-B and WPC-AL flags improved the detection capability of the integrated pathway, while pathological cases among WPC-negative samples were identified through additional institution-specific smear-review criteria.

Although the integrated workflow may support the optimization of smear-review allocation when interpreted together with independent institutional review criteria, these findings should be interpreted within the context of our laboratory setting and should not be directly extrapolated to the entire routine CBC population or to laboratories applying different smear-review criteria.

Further multicentre studies are required to validate these findings in different clinical settings and to define optimal WDF and WPC flag thresholds through simulation analyses, including evaluations of diagnostic performance, smear-review rates, turnaround time, and resource utilization.

## Figures and Tables

**Table 1:** Cross-tabulation between WDF-B/AL flag results and morphological classification. Morphological examination of peripheral blood smears was used as the reference standard for the identification of blasts and atypical lymphocytes.

Morphological Analysis	WDF Negative	WDF-B/AL Positive	Total	%
Negative	1670	1747	3417	75.41
Blasts	109	446	555	12.25
Atypical lymphocytes	22	537	559	12.34
Total	1801	2730	4531	100.00
%	39.75	60.25	100.00	

**Table 2:** Contingency table comparing the WDF-B/AL flag with morphological examination of peripheral blood smears (reference standard) for the detection of blasts and atypical lymphocytes.

Morphological Classification	WDF-B/AL Positive	WDF-B/AL Negative	Total
Morphologically positive for blasts or atypical lymphocytes	983 (TP)	131 (FN)	1114
Morphologically negative for blasts or atypical lymphocytes	1747 (FP)	1670 (TN)	3417
Total	2730	1801	4531

**Table 3:** Diagnostic performance of the WDF-B/AL flag for the detection of blasts and abnormal lymphocytes using morphological examination as the reference standard.

Metric	Value	95% CI
Sensitivity (%)	88.24	86.20–90.07
Specificity (%)	48.87	47.18–50.56
Negative Predictive Value (NPV, %)	92.73	91.43–93.88
Positive Predictive Value (PPV, %)	36.01	34.20–37.84
Accuracy (%)	58.55	57.10–59.99
Positive Likelihood Ratio (LR+)	1.73	—
Negative Likelihood Ratio (LR−)	0.24	—
Area Under the ROC Curve (AUC)	0.75	—
Youden-derived cut-off	130	—
Sensitivity at the Youden-derived cut-off (%)	75.1	—
Specificity at the Youden-derived cut-off (%)	63.4	—

Abbreviations: AUC, area under the receiver operating characteristic curve; CI, confidence interval; LR+, positive likelihood ratio; LR−, negative likelihood ratio; NPV, negative predictive value; PPV, positive predictive value.

**Table 4:** Cross-tabulation between WPC flag categories and morphological findings among WDF-positive samples.

Morphological Classification	WPC Negative	WPC-B	WPC-AL	WPC-B + AL	Total	%
Negative	298	436	981	32	1747	63.99
Blasts	53	219	67	107	446	16.34
Atypical lymphocytes	19	23	477	18	537	19.67
Total	370	678	1525	157	2730	100.00
%	13.55	24.84	55.86	5.75	100.00	

Table legend. Distribution of WPC flag categories according to morphological classification in samples positive for the WDF-B/AL flag.

**Table 5:** Diagnostic performance of the WPC-B flag for the detection of blast cells using morphological examination as the reference standard.

Morphological Classification	WPC-B Positive	WPC-B Negative	Total
Morphologically positive for blasts	326 (TP)	120 (FN)	446
Morphologically negative for blasts	509 (FP)	1775 (TN)	2284
Total	835	1895	2730

Abbreviations: TP, true positive; FN, false negative; FP, false positive; TN, true negative. Table legend. Contingency table comparing the WPC-B flag with morphological identification of blast cells.

**Table 6:** Contingency table for the WPC-AL flag in the detection of morphologically confirmed atypical lymphocytes.

Morphological Classification	WPC-AL Positive	WPC-AL Negative	Total
Morphologically positive Atypical lymphocytes	495 (TP)	42 (FN)	537
Morphologically negative Atypical lymphocytes	1187 (FP)	1006 (TN)	2193
Total	1682	1048	2730

Abbreviations: TP, true positive; FN, false negative; FP, false positive; TN, true negative. Table legend. Diagnostic performance matrix of the WPC-AL flag using peripheral blood smear morphology as the reference standard for atypical lymphocytes identification.

**Table 7:** Diagnostic performance of the WPC-B and WPC-AL flags for the detection of blasts and abnormal lymphocytes, respectively.

Metric	WPC-B Value	95% CI	WPC-AL Value	95% CI
Sensitivity (%)	73.09	68.72–77.16	92.18	89.57–94.31
Specificity (%)	77.71	75.95–79.41	45.87	43.77–47.99
Negative Predictive Value (NPV, %)	93.67	92.48–94.72	95.99	94.62–97.10
Positive Predictive Value (PPV, %)	39.04	35.72–42.44	29.43	27.26–31.67
Accuracy (%)	76.96	75.33–78.53	54.98	53.09–56.86
Positive Likelihood Ratio (LR+)	3.28	—	1.70	—
Negative Likelihood Ratio (LR−)	0.35	—	0.17	—
Area Under the ROC Curve (AUC)	0.81	—	0.77	—
Youden-derived cut-off	70	—	210	—
Sensitivity at the Youden-derived cut-off (%)	77.1	—	65.2	—
Specificity at the Youden-derived cut-off (%)	75.2	—	77.1	—

Abbreviations: AUC, area under the receiver operating characteristic curve; CI, confidence interval; LR+, positive likelihood ratio; LR−, negative likelihood ratio; NPV, negative predictive value; PPV, positive predictive value.

**Table 8:** Diagnostic performance matrix of the aggregated WPC channel (WPC-B OR WPC-AL) for the detection of blasts and abnormal lymphocytes.

Morphological Classification	WPC Positive (WPC-B OR WPC-AL)	WPC Negative	Total
Morphologically positive for blasts or atypical lymphocytes	911 (TP)	72 (FN)	983
Morphologically negative for blasts or atypical lymphocytes	1449 (FP)	298 (TN)	1747
Total	2360	370	2730

Abbreviations: TP, true positive; FN, false negative; FP, false positive; TN, true negative. Table legend. Contingency table of the aggregated WPC response, considering the channel positive when either the WPC-B or WPC-AL flag was activated.

**Table 9:** Diagnostic performance of the aggregated WPC channel (WPC-B OR WPC-AL) for the detection of blasts and abnormal lymphocytes.

Metric	Value	95% CI
Sensitivity (%)	92.68	90.80–94.23
Specificity (%)	17.06	15.32–18.91
Negative Predictive Value (NPV, %)	80.54	76.13–84.45
Positive Predictive Value (PPV, %)	38.60	36.63–40.60
Accuracy (%)	44.29	42.41–46.17
Positive Likelihood Ratio (LR+)	1.12	—
Negative Likelihood Ratio (LR−)	0.43	—

Abbreviations: CI, confidence interval; LR+, positive likelihood ratio; LR−, negative likelihood ratio; NPV, negative predictive value; PPV, positive predictive value. Table legend. Diagnostic performance of the aggregated WPC response, considering the channel positive when either the WPC-B or WPC-AL flag was activated.

## Data Availability

The data presented in this study are available on request from the corresponding author due to subject to approval by the hosting institution and in accordance with applicable institutional policies and data protection regulations.

## Abbreviations

The following abbreviations are used in this manuscript:

WDF	White Blood Cell Differential channel
WPC	White Precursor Cell channel
CBC	Complete blood count
WDF-B/AL	White Cell Differential-“Blasts/Abnormal lymphocytes?” flag
WPC-B	White Precursor Cell-“Blasts?” flag
WPC-AL	White Precursor Cell-“Abnormal lymphocytes?” flag
TP	True Positives
FP	False Positives
TN	True Negatives
FN	False Negatives
PPV	Positive Predictive Value
NPV	Negative Predictive Value
LR+	Positive Likelihood Ratio
LR−	Negative Likelihood Ratio
ROC	Receiver Operating Characteristic
AUC	Area Under the Curve
DI-60	Digital Cell Imaging Analyzer
Q-value	Quantitative flag value generated by the analyzer
